# A Feeding Induced Switch from a Variable to a Homogenous State of the Earthworm Gut Microbiota within a Host Population

**DOI:** 10.1371/journal.pone.0007528

**Published:** 2009-10-20

**Authors:** Knut Rudi, Kristin Ødegård, Tine Therese Løkken, Robert Wilson

**Affiliations:** 1 Hedmark University College, Hamar, Norway; 2 Matforsk, Norwegian Food Research Institute, Ås, Norway; University of Wisconsin-Milwaukee, United States of America

## Abstract

**Background:**

The distribution pattern of the earthworm gut microbiota at the host population level is of fundamental importance to understand host-microbiota interactions. Our current understanding of these interactions is very limited. Since feeding represents a main perturbation of the gut microbiota, we determined the effect of a single dose of feed on the microbiota associated with an earthworm population in a simulated microenvironment.

**Methodology:**

Earthworms were sampled 0, 1 and 7 days after feeding. We determined the overall composition of the earthworm-associated microbiota by 16S rRNA gene cloning and sequencing. Based on the 16S rRNA gene data we constructed quantitative PCR's (Q-PCR) for the seven most dominating bacterial groups.

**Principal Findings:**

Q-PCR revealed low density and highly variable microbiota among the earthworms before feeding, while a high-density homologous microbiota resulted from feeding. We found that the microbiota 1 day after feeding was more equal to the microbiota after 7 days than before feeding. Furthermore, we found that the gut microbiota was very distinct from that of the bedding and the feed.

**Significance:**

The homogenous population response represents fundamental new knowledge about earthworm gut associated bacteria.

## Introduction

The gastrointestinal tract (gut in popular terms) with its microbiota is one of the most important metazoan (animal) organs [1]. The gut is mainly responsible for extracting energy from ingested food. There is an intimate interaction between gut bacteria, having the metabolic capacity to break down polysaccharides or energy sources the host can not directly utilize. A tremendous challenge to the host, however, is to differentiate beneficial from harmful bacteria, since the gut is in direct contact with the environment through ingested food [2]. Despite their importance, we still do not know the general mechanisms governing the transmission and persistence of gut bacteria within a host population [3], [4].

Invertebrates such as earthworms mainly utilize the gut bacteria for the same purposes as vertebrates, for provision of metabolic capacities and protection against pathogens [5], [6], [7], [8], [9], [10]. The importance of earthworms in organic transformations was already recognized by Darwin [11] long before the discovery of bacterial interactions [7]. Since earthworms are in intimate interaction with bacteria, earthworms need an efficient immune system differentiating beneficial from harmful bacteria. Earthworms have both general antimicrobial mechanisms, and selective mechanisms targeting potentially harmful bacteria [12].

There have been numerous studies on the earthworm microbiota [5], [6], [7], [8], [9], [13], [14], [15]. Very little, however, is known about the distribution pattern of bacteria within earthworm populations. In particular, fundamental questions about origin and spread of gut bacteria within host populations remain unanswered. Addressing these questions will be important both to understand soil microbiota ecology and general principles of host microbiota interactions.

The main transfer of bacteria within an earthworm population occurs through feeding and excretions (Drake and Horn 2007). The aim of the current work was therefore to determine the effect of feeding on the microbiota associated with a population of earthworms in a simulated microenvironment.

We used the epigeic earthworm *Eisenia hortensis* (European nightcrawler) as a host population model. The rationale for using surface-feeding earthworms is that they have a diverse feeding repertoire consisting of plant and animal material [16]. We present empirical evidence that there is a rapid and consistent change of the microbiota in the gut at the host population level after feeding. Our results also showed that feeding significantly reduces individual variation in the microbiota.

## Materials and Methods

### Experimental material and setup

Earthworms of the species *Eisenia hortensis* were purchased from a commercial supplier (Fibe, Överkalix, Sweden). Approximately 100 paradiapaused earthworms were starved under low moisture conditions at 18°C for 14 days prior to the experiment. This was done to ensure emptying of the gut [17]. We simulated the natural environment of earthworms using bedding consisting of a mixture of an organic fiber and Canadian sphagnum peat moss with a natural microbiota (Magic Products, Inc, Amherst Junction, USA). The bedding was kept at 80% moisture in a 14″×20″×7″ aerated container at a constant temperature of 18°C. The earthworm population was given a single feeding of 50 g Magic® Worm Food (Magic Products). This corn-based feed contains a combination of 32 different proteins, fats, minerals, vitamins, and carbohydrates which are essential to the earthworm. The main composition of the feed was approximately 12% protein, 1% fat, 81% carbohydrates and 6% fiber (www.magicproducts.com). The feed was given as water slurry, and was consumed within approximately 2 days.

Five earthworms were collected at each of three time points at day 0, 1 and 7 after feeding, in addition to an earthworm that was sampled directly after purchase from a population of approximately 100 earthworms. The collected earthworms were rinsed in distilled water, killed in 70% ethanol, and then immediately frozen at −80°C. In addition to the earthworms, approximately 20 g of the feed, the original bedding (pre-experiment) and the bedding at day 7 (post-experiment) were collected and stored at −80°C.

Finally, an experiment was conducted to investigate the non-earthworm growth of spoilage bacteria in the feed. Five ml feed was mixed with 1 ml bedding, and water was added to a total volume of 15 ml with a 35 ml headspace of air. This mixture was incubated at 18°C with slight agitation in a tight tube with air flushing after 1 day, and samples were collected at the same time-points as for the earthworm experiment.

### Sample preparation and DNA purification

**Figure 1:**
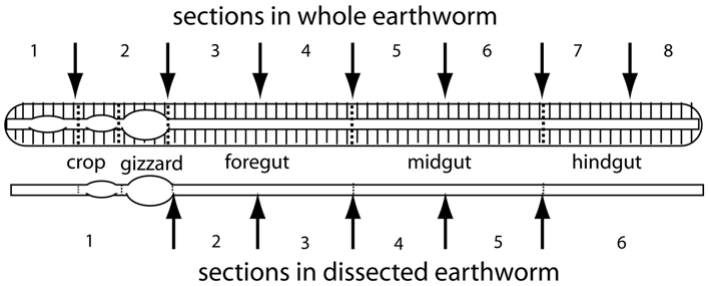
Schematic representation of earthworm dissection schemes. We analyzed longitudinal segments of both whole and gut-dissected earthworms. Whole earthworms were divided into 8 segments, while the gut-dissected earthworms were divided into 6 segments.

Earthworms were dissected following two different schemes (Fig. 1). The dissections were performed in a sterile environment using the Deluxe Anatomy Pre-Med Dissecting Kit (Indigo Instruments, Unit I, Waterloo, USA), following general guidelines for earthworm dissection. The rationale of the dissection schemes is to describe the longitudinal distribution of earthworm bacteria. For practical reasons, whole earthworms were divided into eight segments while the gut-dissected earthworms were divided into six. The dissected samples were transferred directly to 96-deep well plates containing 700 µl 4 M guanidine thiocyanate (GTC) and 200 mg acid-washed glass beads (212–300 µm, Sigma, St. Louis, USA). The samples were then mechanically lysed using a bead beater (Mini-Beadbeater, Biospec Products, Bartlesville, USA) two times for 5 minutes each. A brief centrifugation was included to pellet debris, with subsequent transfer of 250 µl of the lysate to fresh tubes. Then, 10 µl Sarkosyl (1%) was added and the samples were incubated at 60°C for 30 min. Finally, 200 µl of the lysate was transferred to a GenoM-96 robot (GenoVision, Oslo, Norway), and a standard DNA purification protocol was followed using 15 µl MagAttract paramagnetic beads (Qiagen, Hilden, Germany), eluting the DNA in 100 µl water.

### Determination of microbiota composition by 16S rRNA gene cloning

We cloned and sequenced the 16S rRNA coding gene using the Bacteria amplicon targeted to generally conserved regions of the 16S rRNA gene (Table 1). The PCR amplification reaction contained 1× Hot Start Buffer (Finnzymes), 0.5 pmol of each primer, 200 µM dNTP mix, 1 U DynaZyme Hot Start DNA Polymerase and 2 µl DNA in a 25 µl PCR reaction. The following amplification program was used: 95°C for 30 s, 60°C for 30 s and 72°C for 45 s. We generally used 30 cycles for the amplification. For samples containing low amounts of bacteria such as the feed and the original bedding (giving a faint band at 30 cycles), we used 35 cycles. Prior to the amplification, the samples were heated to 94°C for 10 min to activate the polymerase, and to denature the DNA. The cloning and DNA sequencing were performed as previously described [18]. The DNA sequences were deposited in GenBank with the following accession numbers: FJ448539-FJ449538.

**Table 1:** Real-time quantitative PCR amplicons used.

Amplicon	Primer pair (F; forward, and R;reverse)	Position^2^	Ampl efficiency	Detection threshold^4^
Earthworm	5′ACGAACGAGACTCTAGCCTGC3′ 1 (F)	1285–1304	0.93	ND
	5′GGGACGTAATCAACGCGAGC3′ 1 (R)	1551–1570		
Bacteria	5′TCCTACGGGAGGCAGCAGT3′ 5 (F)	340–358	0.89	ND
	5′GGACTACCAGGGTATCTAATCCTGTT3′ 5 (R)	781–806		
Proteob I	5′TCCTACGGGAGGCAGCAGT3′ (F)	340–358	0.91	ND
	5′GGTTGAGCCCGGGGATTTCACATCTGTC3′ 1 (R)	597–624		
Proteob II	5′TCCTACGGGAGGCAGCAGT3′ (F)	340–358	0.81	−5
	5′TGCTTATTCTTACGGTACCGTCATGC3′ 1 (R)	477–502		
Proteob III	5′GTTGGTGTCTTGACGTTACCGAC3′ 1 (F)	470–492	0.66	−4
	5′ACTTAACAAACCACCTACGCGC3′ 1 (R)	577–598		
Bacteroid I	5′GTGCGCGAGAAATTGAATGTACCTGGC3′ 1 (F)	∼464–490^3^	0.84	−7
	5′GCCTACCTCATCAACACTCAAGTCC3′ 1 (R)	648–672		
Actinob I	5′TCCTACGGGAGGCAGCAGT3′ 1 (F)	616–631	0.90	−5
	5′GGACATGCCCAGAGAACCGC3′ 1 (R)	741–756		
Actinob II	5′TCCTACGGGAGGCAGCAGT3′ 1 (F)	616–631	0.82	−5
	5′GGTGTTCCTCCTGATATCTGCGCATTC3′ 1 (R)	741–756		
Actinob III	5′GCTTGCTTCCGATACGGGC3′ 1 (F)	629–647	0.82	ND
	5′CGCTCCTCAGCGTCAGGTAATTC3′ 1 (R)	743–765		
Archaea	5′TCCAGGCCCTACGGG3′ 6 (F)	348–362	ND	ND
	5′YCCGGCGTTGAMTCCAATT3′ 6 (R)	958–939		

1 Constructed in this work.

2 Position is relative to *Lumbricus terrestris* 18S rRNA for the Earthworm amplicon, while the Bacteria, Proteob I, II and III, Bacterioid I, and Actinob I, II and III are numbered relative to *E. coli* 16S rRNA, and Archaea relative to archaeal 16S rRNA [30].

3 Approximate positions due to low DNA sequence identity.

4 The detection threshold is a log approximation relative to total bacterial DNA determined by the Bacteria amplicon. ND – not determined.

5 From [31].

6 From [30].

The overall microbiota composition based on 16S rRNA gene sequence data was determined both by the AIBIMM approach [19], and by using the Ribosomal Database Project II (RDP-II) hierarchical classifier with default settings [20]. AIBIMM is based on alignment-independent classification in a coordinate system, while RDP-II uses a predefined model for classification. The clone frequency data in different libraries were compared by density distribution clustering tree analyses of the AIBIMM data [18]. Briefly, the density approach involves the transformation of the AIBIMM taxa coordinate data into density distributions, with subsequent comparisons of densities in the different libraries. The dendrogam approach, on the other hand, involved calculating all pair-wise Euclidian distances for the three first AIBIMM PC's, and then to construct a clustering tree based on the divisive hierarchical clustering algorithm (S-plus 7.0, Insightful Corp., Seattle, Washington, USA).

### Construction of selective 16S rRNA gene targeted amplicons

The selectivity of the amplicons used is normally evaluated based on a set of pure cultures. Obviously, such evaluations will not be relevant for a soil or earthworm environment. We therefore chose to use our own clone library for evaluation of the specificity of the amplicons. In this way we ensured the selectivity of our amplicons with respect to the main bacterial groups expected in our samples.

The criteria for primer construction required that the eight three-prime nucleotides in the primer were conserved among all the target organisms, and that the total number of mismatches in the primer should not exceed three for target organisms. With respect to the discrimination of non-target organisms, the primers were constructed either with a discriminatory cytosine in the three-prime end of the primer, or at least three mismatches for the non-target organisms in the 10 three-prime nucleotides of the primer. Primers were constructed with a Tm of approximately 60°C, as determined using the nearest neighbor method for Tm calculations [21].

### Determination of microbiota composition by real-time quantitative PCR

We used the DyNAmo™ HS SYBR® Green qPCR Kit (Finnzymes, Espoo, Finnland) for the real-time PCR analyses. We used 10 µl reaction volumes containing a 1× master mix, 2 pmol of each primer and 1 µl template. The following cycling conditions for the real-time PCR were employed: 95°C for 30 s, 63°C for 30 s and 72°C for 1 min for all the amplicons. We included a denaturation/activation step at 94°C for 10 min prior to the amplification, and a melting curve analysis after the amplification.

Amplicons for SYBR-green-based real-time quantitative PCR were constructed based on 16S rRNA gene signature sequences, while the earthworm-specific amplicon was constructed based on the 18S rRNA gene using Primer Express 3.0 (Applied Biosystems, Foster City, USA). The amplification efficiencies were determined from the log linear part of the amplification curves [22], while the specificity of the primers was determined by melting-curve analyses, and by screenings of the cloned 16S rRNA gene sequences by real-time quantitative PCR.

Traditionally, quantifications have been expressed relative to the wet- or dry weight of the material analyzed. Since most of the earthworm gut content is either feed or soil, weight measurements are highly dependent on the amount of the ingested material. We therefore chose to use earthworm DNA as an internal reference for the whole earthworm longitudinal sections. Differences in the ratio between earthworm and bacterial DNA between individuals are likely due to differences in bacterial load. For the gut-dissected samples, on the other hand, we quantified the relative composition of bacteria using the Bacteria amplicon as a reference.

We analyzed the real-time quantitative PCR data by multivariate regression using partial least square regression (PLSR) [23] with time after feeding as the predictor variable and relative quantity of bacteria as the response variable. Fuzzy clustering using the default parameters in S-plus 7.0 (Insightful, Seattle, USA) was used to determine group structure in the loading plot. The loading plot represents the importance of the predictor variable (days after feeding) with respect to the response variable (relative quantity of bacteria). The reason for using fuzzy clustering and not crisp clustering is to reveal uncertainties in the cluster assignments [24].

The analysis of variance in the bacterial distribution between starved and fed earthworms was performed by first parameterizing the distribution in the individual earthworms for each bacterial group investigated by real-time PCR. We used three parameters by fitting a second order polynomial trend line to the spatial distribution for the eight segments analyzed (Microsoft Excel 2003 SP2, Microsoft Corp., Redmond, USA). For each bacterial group we then determined the variance for the three parameters for the earthworms in the day 0 and day 7 categories, respectively. Subsequently, the binary (day 0 and day 7) ranges of the variance for all the analyzed parameters were determined. Finally, the Wilcoxon rank-sum test was used to determine if the range for the variance for the day 0 and day 7 categories were significantly different (S-plus 7.0).

## Results

### Phylogroup-specific real-time quantitative PCR's

**Figure 2:**
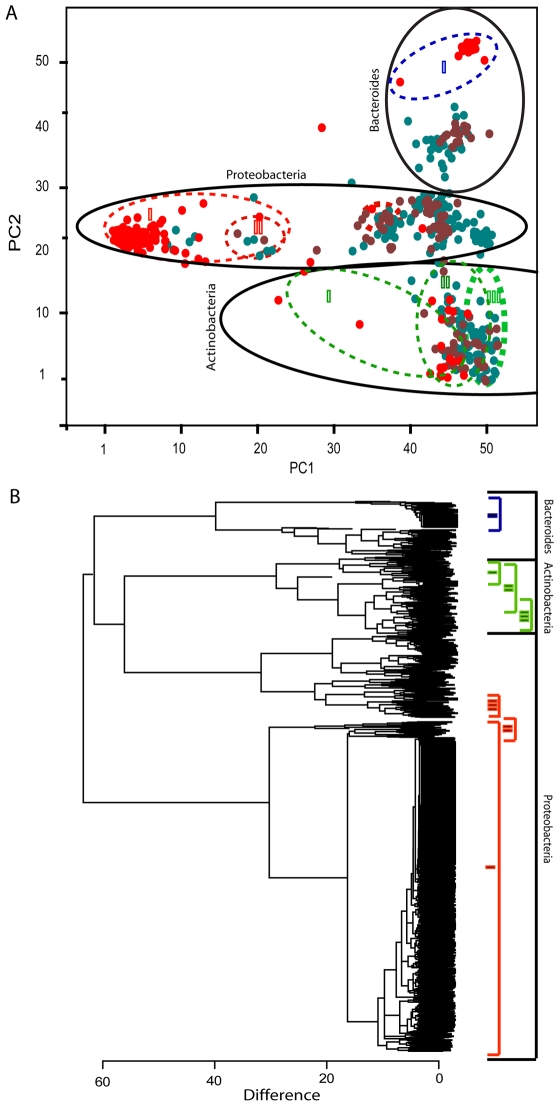
Total bacterial diversity associated with earthworms determined by 16S rRNA gene clone library analyses. PCA plot (A) and dendrogram (B) representation of all the bacterial 16S rRNA gene sequences (n = 1000) determined in this work. In the PCA plot, the major bacterial groups identified are marked with solid lines, while the selectivity of the amplicons used (Table S1) are marked with color-code stippled lines. Corresponding color-coding is given for the dendrogram. The PCA color-coding represents the respective libraries; red – whole earthworm, brown – gut-dissected earthworm, and blue –bedding/feed. The PCA axis numbering represents the respective AIBIMM coordinates.

We investigated the microbiota associated with two datasets in order to construct phylogroup-specific real-time quantitative PCR's. The first subset (n = 333) represents a comparison between the microbiota in bedding/feed and the midgut of earthworms post experiment (day 7), while the other subset (n = 667) represents the longitudinal distribution of bacteria in the single earthworm sampled just after purchase. Taking both datasets together, the microbiota was mainly composed of *Proteobacteria* (n = 705), *Actinobacteria* (n = 134) and *Bacteroidetes* (n = 97), with a minor importance of *Firmicutes* (n = 5), *Verrucomicrobia* (n = 2), *Planctomycetes* (n = 1) and Genera_incertae_sedis_TM7 (n = 3) (Table S1). The clustering analyses showed that the microbiota was composed of relatively distinct clusters (Fig. 2). We estimated that new clones should, with a probability of approximately 90%, be within the already defined phylogroups. This estimate was based on the frequency of single clone phylogroups in our dataset. Interestingly, there was a linear relationship between the log of the frequency and the log of the rank of the density distribution (Fig. S2). Details about the specific microbiota distributions are described in Text S1 and in Fig. S3.

Based on the clustering patterns, we constructed seven real-time quantitative PCR amplicons covering approximately 90% of the described microbiota diversity. The phylogroups covered by the constructed amplicons are shown in Figure 2. The primer sequences are presented in Table 1.

We used a two-step process in evaluating the constructed amplicons. The first evaluation was based on the specificity, sensitivity and reproducibility of the amplicons. This was done by evaluating the amplification products from the earthworm samples by quantitative PCR, agarose gel electrophoresis, and melting curve analyses. Quantitative PCR showed that replicated PCR's for the same DNA generally deviated with less than one PCR cycle. The criteria for specificity were that only a single band of expected size, and a single peak with expected melting temperature should be detected. Based on these empirical evaluations we determined the detection limit for each amplicon. This information is given in Table 1.

In the second part of the evaluation, we determined the selectivity of the amplicons. These evaluations are summarized in Table 2. Our evaluations were based on a 16S rRNA gene reference library composed of 92 clones. The sequenced clones are represented with coordinates that indicate the phylogenetic placement in Fig. 2. The evaluation criterion was the amplification of the selective amplicons relative to the Bacteria amplicon. We did not evaluate the amplicon Bacteroid I because the target bacteria for this amplicon were in low abundance in the main sample set analyzed.

**Table 2:** Evaluation of PCR amplification selectivity^1^.

		1	2	3	4	5	6	7	8	9	10	11	12
Coordinates	A				46, 3		43, 28						41, 24
	B	44, 20			9, 25		12, 24	39, 26				44, 39	
	C	45, 24	52, 12	43, 40		41, 35	44, 29					11, 23	
	D	46, 38		47, 8			46, 24		46, 45			45, 5	
	E	41, 30	38, 26					44, 27			43, 31		
	F	47, 12			38, 27			47, 25					
	G	48, 5				33, 22					44, 25	46, 26	
	H			44, 37	36, 27		39, 28		38, 26				38, 24
Proteob I	A	−0.28	−6.62	−4.93	−5.25	−3.87	−3.79	−4.7	−4.7	−0.03	0.41	−5.5	−5.37
	B	−4.29		−3.15	0.66	−5.08	−0.34	−5.13	−2.65	−3.87	−2.95	−5.9	−4.86
	C	−5.27	−5.45	−5.62	−3.77	−4.96	−6.09	−4.7	−4.98	−5.57	−2.23	−0.16	−3.46
	D	−5.32	−6.36	−5.44	−4.67	−4.32	−6.07	−4.5	−3.92	−4.86	−3.39	−5.08	−3.52
	E	−4.11	−5.47	−5.32	−2.52	−4.67	−5.75	−4.77		−4.97	−2.81	−4.37	−5.76
	F	−5.65	−5.34	−5.25	−3.86	−5.03	−5.23	−3.92	−3.46	−0.09	−4.81	−4.37	−4.16
	G	−5.46	−5.89	−5.76	−3.25	−5.52		−3.91	1.46	−3.59	−4.47	−5.44	−5.45
	H	−5.7	0	−5.42	−3.26		−4.17	−0.44	−4.88	−3.62	−3.2	−0.91	
Actinob III	A	−7.12	−5	−6.9	−7.1	−3.13	−7.05	−6.22	0.5	−8.36	−6.93	−7.64	−3.13
	B	−4.48	−6.93	−6.81	−8.13	−6.62	−7.36		−5.28	−6.8	−8.33	−7.4	−7.54
	C	−6.56	−1.83	−7.07	−6.32	−6.75		−5.33	−2.22	0.02	−7.01	−7.4	−7.09
	D	−6.54	−7.09	−7.87	−6.91	−6.75	−7.21	−7.41	−5.39	−5.9	−4.42	−6.79	−7.27
	E	−8.55	−5.99	−6.92	−6.95	−7.05	−7.25	−6.8		−6.85	−7.94	−7.01	−6.27
	F	−7.54	−4.64	−7.01	−6.73	−7.52	−6.8	−8.19	−6.29	−6.3	−7.13	−7.03	−4.71
	G	−5.63	−7.07	−6.85	−7.71	−7.42		−7.93	−5.67		−6.76	−6.67	−4.92
	H	−6.37	−6.29	−7.72	−6.78		−7.53	−7.94	−7.27	−7.09	−6.98	−7.6	
Actinob II	A	−3.04	0.2	−0.71	0.06	−4	−2.5	−5.76	0.34	−2.47	−2.51	0.33	−4.85
	B	−0.68	−4.73	0.51	−2.37	0.06	−3.01	−2.24	1.15	−4.55	−4.99	−6.47	−4.92
	C	−1.43	0.31	−5.96	−4.7	−5.69	−6.53	0.19	0.88	0.07	−5.15	−2.48	−5.65
	D	−5.44	0.23	0.05	−0.65	−0.37	−5.66	0.13	−5	−0.05	−5.57	0.36	−5.23
	E	−5.19	−3.22	−2.25	−5.25	0.27	0.01	−6.14		0.27	−4.44	−0.09	0.13
	F	−0.18	0.61	0	−1.88	−0.64	−1.88	−3.19	1.1	−2.41	−0.04	−2.18	−0.09
	G	−0.49	0.1	−5	−4.91	−3.61	−5	0.12	−1.2	−5.64	−5.51	−4.85	−0.13
	H	−5	−2.6	−5	−4.06		−5.64	−3.87	−2.14	−4.65	−5	−2.81	
Proteob III	A	−4	−4	−5.91	−4	−6.84	−5.95	−4	−4	−4	−4	−6.02	−5.64
	B	−4	−6.04	−4	−4	−6.15	−4	−4	−4	−4	−5.59	−6.45	−5.54
	C	−4	−4	−4	−4	−4	−4	−4	−4	−6.66	−4	−4	−4
	D	−4	−5.9	−4	−6.54	−6.87	−4	−5.77	−2.87	−4	−4	−4.52	−4
	E	−5.71	−3.8	−4	−4	−5.66	−6.82	−5.75		−4	−4	−5.84	−4
	F	−4	−4	−4	−5.24	−6.09	−5.24	−4	−4	−6.06	−4	−6.02	−4
	G	−6.09	−5.55	−5.82	−5.48	−5.69	−4	−6.42	−4.33	−6.64	−4	−4	−6.07
	H	−4	−5.23	−5.81	−4		−5.65	−2.41	−4.78	−4	−4	−4	
Proteob II	A	−3.25	−4.94	−3.44	−5.48	−5.81	−6.02	−4.85	−5.33	−5	−5	−4.94	−6.51
	B	−5.02	−5.83	−5.32	−2.82	−4.92	−3.06	−6.87	−3.34	−5.04	−7.2	−6.66	−6.3
	C	−5.48	−4.45	−4.18	−5.27	−5.83	−6.26	−4.66	−3.95	−4.64	−6.44	−3.13	−5.43
	D	−3.84	−6.09	−5.84	−5.66	−4.89	−6.2	−5.29	−5	−4.9	−6.42	−5	−4.97
	E	−4.79	−5	−3.02	−4.75	−4.62	−4.9	−6.16		−4.99	−6.19	−6.4	−5
	F	−5.85	−4.54	−5.06	−5.15	−4.83	−5.36	−5.49	−3.59	−3.51	−5.87	−1.54	−4.75
	G	−4.59	−4.38	−5.48	−4.91	−6.21	−5	−4.86	−1.87	−7.18	−6.15	−5.73	−5.15
	H	−4.77	−3.23	−6.07	−6.14		−6.2	−5.24	−5	−5.62	−5.97	−3.87	
Actinob I	A	−3.92	0.51	−2.35	−1.32	−5.36	−5.27	−6.42	0.69	−4.65	−5.59	−4.14	−5.59
	B	−3.11	−3.65	−3.38	−4.87	−0.79	−5.69	−4.7	0.77	−3.86	−4.02	−6.08	−4.2
	C	−4.21	0.01	−6.21	−3.11	−5.72	−4.48	0.03	1.19	−0.19	−5.52	−5.31	−5.2
	D	−3.3	−0.95	−3.26	−3.03	−0.96	−4.17	0.31	−3.48	0.06	−4.05	−1.32	−4.07
	E	−3.67	−4.17	−5.45	−4.35	0.1	−4.62	−4.45		−0.9	−4.31	−2.45	−0.5
	F	−1.78	0.8	−3.46	−6.85	−3.68	−4.91	−5.11	1.13	−4.83	−5.56	−4.67	−2.54
	G	−0.26	−3.88	−4.95	−3.49	−3.89	−5	−2.64	−3.28	−6.15	−4.78	−6.39	−2.56
	H	−2.63	−4.07	−5	−5.87		−6.19	−5.51	−5.19	−4.63	−4.32	−6.43	

1 The table replica information for an 8×12 matrix of cloned 16S rRNA genes. The template was PCR-amplified plasmid DNA in a 10^−3^× concentration. The first replica of the matrix shows the position (PC1, PC2) of a selection of the samples relative to the coordinates in Figure 2. The rest of the matrix replica shows the amplification for the amplicons shown in the first column. The numbers are the log_10_ of signals relative to the Bacteria amplicon.

The Bacteria amplicon showed relatively uniform amplification with a variation of approximately one to two cycles. Generally, the selective amplicons exhibited a good discrimination between target and non-target bacteria (Table 2). There was, however, a relatively large overlap between Actinob I and Actinob II, and Actinob II and III, while Proteob II represents a subset of Proteob I. Unfortunately, no target bacteria for Proteob III were present in the test set, but from our evaluation we can conclude that the amplicon showed a good selectivity with respect to non-target bacteria.

There was good correspondence between the criteria used for construction of the selective PCR amplicons and the actual selectivity, but there was some divergence in the selectivity between the theoretical and observed selectivity for Actinob I. The observed selectivity was broader than the theoretical selectivity.

### Spatial, individual and temporal variance in the distribution of the microbiota

We investigated the longitudinal distribution of the seven main bacterial phylogroups described above using real-time quantitative PCR. We analyzed both whole and gut-dissected earthworms (see Fig. 1 for dissection schemes), in addition to bedding, feed, and non-earthworm-associated growth of bacteria in the feed. We also investigated the distribution of *Achaea* in a subset of the samples.

**Figure 3:**
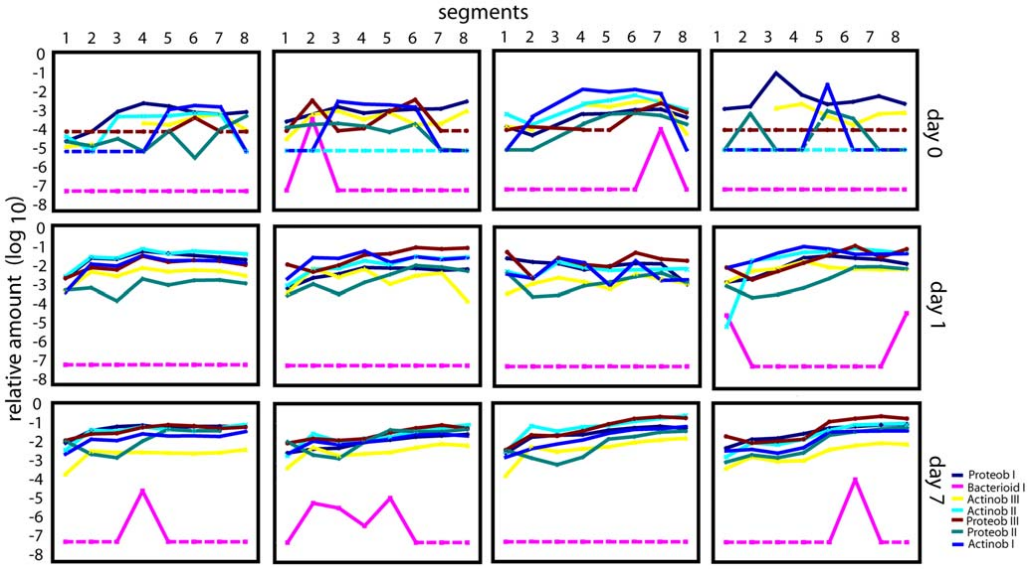
Longitudinal distribution of bacteria in earthworms with respect to time after feeding. Each panel represents the analysis of a single earthworm. The distribution of bacteria was determined by real-time PCR, quantifying the amount of 16S rDNA relative to total earthworm DNA (segmentation as described in Fig. 1). The line color indicates the different bacterial groups – shown in the figure. The stippled lines indicate that the given bacterial group was below the detection limit.

**Figure 4:**
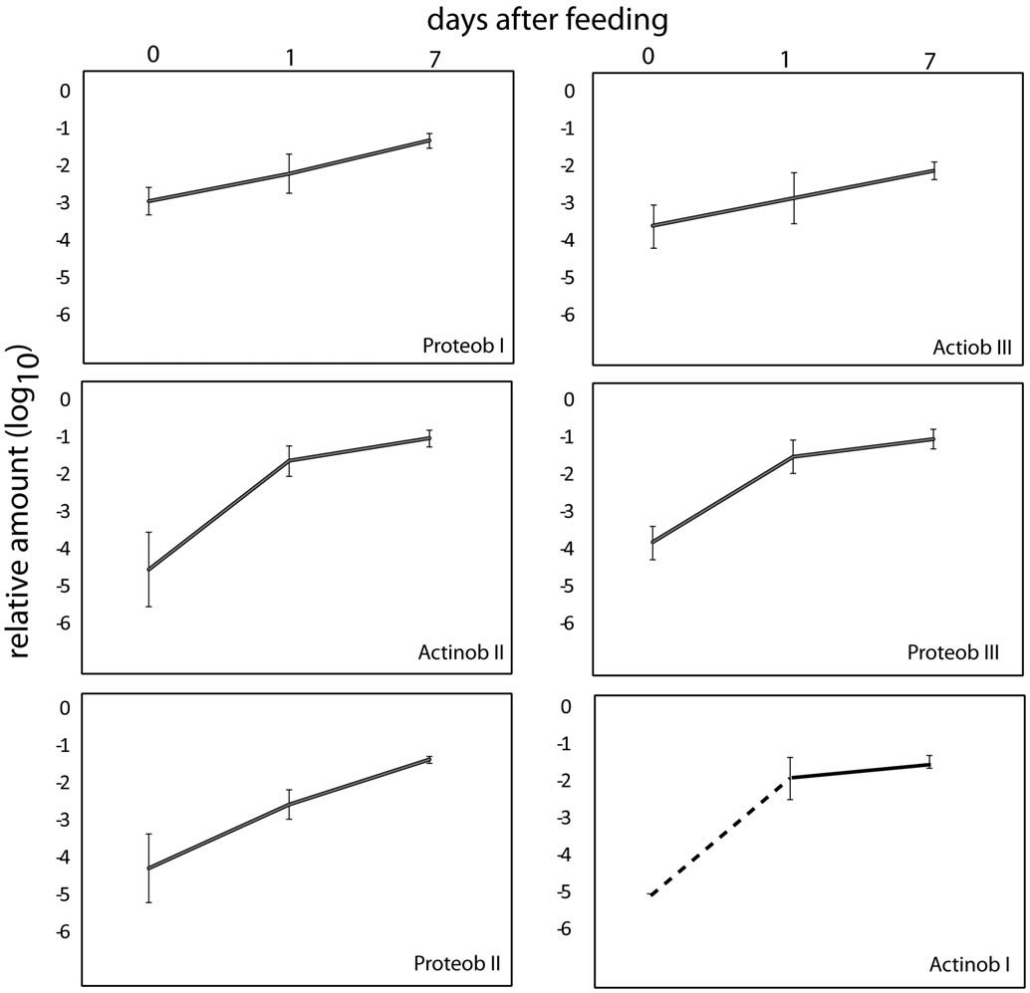
Temporal development of bacteria in the earthworm hindgut. Each panel shows the bacterial group averages and standard deviations for segment 8 based on the data presented in Figure 3.

For the overall earthworm-associated microbiota we found a relatively large individual and spatial variance in the distribution of bacteria in the starved earthworms (Fig. 3). The only two bacterial groups with a relatively constant level in the samples analyzed were Proteob I and Actinob III. Proteob I represents mainly bacteria related to the symbiont *Acidiovorax*, while Actinob III is related to *Propionibacteria.* There was a remarkably rapid and consistent response in the microflora with respect to feeding (Fig. 3 and 4). Using fuzzy cluster analyses based on the PLSR data we showed that earthworms cluster according to the day after feeding, with day 1 and 7 being more similar than day 0 (Table 3). The positive PLSR loadings showed an increase for the overall bacterial content, with Actinob I and II, and Proteob II and III increasing most (Fig. S4).

**Table 3:** Fuzzy clustering of earthworms based on PLSR regression score plot.

True category (day)	cat #1	cat #2	cat # 3	crisp	cat # 1	cat # 2	crisp
0	0.98	0.01	0.01	cat #1	0.98	0.02	cat #1
0	0.98	0.01	0.01	cat #1	0.98	0.02	cat #1
0	0.63	0.22	0.15	cat #1	0.80	0.20	cat #1
0	0.94	0.03	0.03	cat #1	0.96	0.04	cat #1
1	0.01	0.93	0.05	cat #2	0.08	0.92	cat #2
1	0.01	0.93	0.06	cat #2	0.07	0.93	cat #2
1	0.07	0.75	0.18	cat #2	0.18	0.82	cat #2
1	0.03	0.76	0.21	cat #2	0.05	0.95	cat #2
7	0.01	0.05	0.94	cat # 3	0.06	0.94	cat #2
7	0.03	0.19	0.79	cat # 3	0.04	0.96	cat #2
7	0.01	0.03	0.97	cat # 3	0.05	0.95	cat #2
7	0.01	0.03	0.96	cat # 3	0.05	0.95	cat #2

1 Three and two fuzzy categories were analyzed, respectively. Membership values are shown for each category. The crisp classification shows the most likely classification.

As seen from Figure 3, there is an apparent temporal reduction in the difference in the bacterial distributions between earthworms. The significance of the reduction in variance was analyzed by fitting a second order polynomial line to the longitudinal distribution of each bacterial group for each individual earthworm analyzed. The variance for the estimated parameters were then determined for the earthworms within the time categories 0 and 7 days. Finally, we used a non-parametric test to determine if there were significant differences in the estimated variances (see Materials and Methods for details). We found a clear reduction in the variance from 0 to 7 days after feeding using the approach described above (p<0.001).

The spatial distribution of bacteria was analyzed by complete dissection of surface/muscle tissue, gut wall, and gut content of all eight longitudinal segments. These analyses showed that the amount of bacteria in the gut content is about one log_10_ higher than for the gut wall, and between one and two log_10_'s than that of the surface/muscle tissue (Fig. S5). In addition, we determined the composition of bacteria in the gut content during the course of our experiment by the phylogroup-selective PCR's. These analyses showed the same main patterns as for the whole earthworms, but with lower relative abundance of Proteob I (Fig. S6). This is probably because these bacteria reside in the earthworm nephridia.

Our final analyses sought to determine the origin of the bacteria found in the earthworm gut. The bedding and feed samples showed a low content of the phylogroups analyzed, and there was no selective enrichment when comparing the bedding samples taken before and after the experiment. The non-earthworm-associated growth of bacteria in the feed showed an approximately 2 log_10_ increase in the total bacterial number during the incubation period, while the level of all the seven earthworm-associated phylogroups were low (<1% relative to the total bacterial population). All samples analyzed also showed a low content of *Archaea* (<1% relative to the total bacterial population).

## Discussion

There has been a long-standing debate of whether bacteria can colonize the earthworm gut [7], [9], or if they only transiently pass through the gut without colonization [6]. Recent evidence, however, points towards the colonization hypothesis. Bacterial colonization has been demonstrated both for the adult [8] and the juvenile [25] earthworm gut. In fact, Davidson and Stahl have demonstrate extensive gammaproteobacterial gut colonization in 22 day old embryos (This is shown in Fig. 6 in their publication). In addition, a set of earthworm gut-associated bacteria was previously identified using a combination of FISH, SSCP and DNA sequencing [9], [15]. In addition, using low resolution tRFLP, significant differences in the microflora between earthworm feed, midgut content and cast were determined, although the authors concluded that an indigenous earthworm microbial community appears unlikely[14]. In our work using high-resolution DNA sequence-based techniques we identified both significant spatial distribution differences in the earthworm gut microbiota, in addition to bacteria that were selectively enriched in the gut. There was also a general increase in the amount of bacteria towards the anterior part of the earthworm, suggesting growth of bacteria through the gut passage. Our results therefore support the gut-associated bacteria hypothesis.

With respect to the ecological relevance of our findings, we have shown a rapid and homogenous change in the gut microbiota at the host population level as a response to feeding. This change was very distinct from that observed for non-earthworm growth of bacteria in the feed. We have also shown that the variance among individuals in the gut microbiota is reduced. A fundamental question with respect to the gut microbiota is what causes underlie the feeding-induced switch in microbiota ecological patterns.

To our knowledge, there are two potential explanations for the observed feeding-induced switch. The first explanation is that the low bacterial density and the large individual variance are due to the feeding regime by the earthworm supplier and not the starvation, and that the switch actually represents the change between two feeding regimes. The second explanation is that the shift is due to the change from a starved to a fed state. The observed shift, however, does not represent a shift in the composition of the microbiota, but rather a shift in abundance and variance. With respect to a change in feeding regimes, we would have expected a shift in the microbiota composition. Therefore we find the second explanation more likely, namely that the starved microbiota is in the non-equilibrium stochastic domain, as defined by De Angelis and Waterhouse [26], thus causing the large variance. This domain is characterized by strong external factors limiting growth, leading to no, or low, interspecies competition and stochastic fluctuations in population densities. Thus, a combination of strict host control and restricted energy sources could be the limiting external factors for bacterial growth in the starved earthworms. The earthworm itself is a potential energy source for bacterial growth. Thus, the bacteria that can metabolize the earthworm must be suppressed, simply because they are potential pathogens. On the other hand, bacteria that can utilize ingested energy sources are suppressed by limited energy supplies. The second explanation is also supported by a simulation showing that low bacterial densities and restricted competition leads to an unstable community with several bacterial types occupying the same niche. Simulation of the fed situation, on the other hand, resembles an equilibrium state with stable community composition, high cell densities and internal competition (see Text S1 and Fig.S1 and S7 for details).

A recent evaluation of the antimicrobial effect of earthworm gut fluid showed survival and growth of a limited number of bacterial species [5]. It has also been shown that the earthworm innate immune system has both specificity and memory [27]. Thus, there is a selective component of the immune system for potential differentiation between beneficial and harmful bacteria. These mechanisms are in accordance with our observations of a homogenous effect of feeding on the microbiota through combined host selection and feed induction of bacterial growth. A particularly interesting question, however, is if the homogenous population response is due to independent selection within each earthworm, or if the response is due to bacterial flux among individuals within the earthworm population so that the niches are not restricted to individual earthworms. Individual responses would imply the presence of endemic gut bacteria, while earthworm population responses would imply earthworm-associated bacteria with the ability colonize the gut, but without strict restriction to the gut environment.

A controversy in earthworm feed utilization is the rapid transit time and low assimilation rates[16]. For *E. hortensis* our empirical observations suggest that the gut was emptied within 24 hours when the earthworms were removed from the bedding. It has been suggested that reingestion of cast could be a mechanism to better utilize feed [28]. Cast reingestion could potentially explain both the homogenous feeding response, and the persistence of the earthworm-associated bacteria. Therefore, of future interest will be to determine the actual exchange rates and mechanisms of bacterial exchange among the individuals in a host population. This will enable more accurate modeling of how the bacteria actually spread in the host population. Ultimately, this knowledge will help us to better understand the interplay between bacteria and hosts in natural ecosystem assemblies [29].

## Supporting Information

Text S1 (0.04 MB DOC)

Table S1 RDPII hierarchical classification of 16S rRNA
(0.23 MB DOC)

Figure S1 Model for computer simulation of bacterial growth. (A) For each generation, the bacterial objects can either divide or die. (B) The decision of division or death is based on cell density-dependent internal competition (bottom-up) of four bacterial groups occupying the same niche. The total cell density within the niche is limited by external factors (top-down).
(5.22 MB TIF)

Figure S2 Density distribution curve for the earthworm-associated microbiota. The log10 of the relative abundance of the phylogroups (squares in Fig. 2) is plotted as a scatter plot with respect to the log10 of the range of the phylogroups. Only phylogroups with a abundance of n = 4 or higher are included due to the reliability of the density determinations. The formula for the regression line is as follows: Abundance (log10)  = −1.2×Range (log10)−0.7, R2 = 0.98.
(2.20 MB TIF)

Figure S3 Longitudinal distribution of a bacterial group significantly overrepresented in the earthworm midgut region. The group is defined by the coordinates 48, 1 in Figure 2, and was overrepresented at the p = 0.05 level. The relative distributions in the eight segments analyzed (see Fig. 1 for reference) are shown.
(2.64 MB TIF)

Figure S4 Regression between days after feeding and longitudinal distribution of bacteria in earthworms. The regression is based on the real-time quantitative PCR data. Results for the first PC are shown, explaining 70% of the variance for the bacterial groups and 58% of the variance for days after feeding data. (A) A score plot showing the relatedness in the microbiota with respect to days after feeding. (B) A loading plot showing which bacterial groups and segments that are important for explaining the overall pattern shown in panel A. The numbers 1 → 8 refers to the segments analyzed.
(7.93 MB TIF)

Figure S5 Spatial distribution of earthworm bacteria. The spatial distribution of bacteria was determined one day after feeding for a single earthworm. Each of the eight segments for the whole earthworm analyses were dissected into three samples: surface/muscle, gut wall and gut content. The quantification of bacteria is expressed relative to the weight of the material analyzed using the Bacteria primer pair.
(3.96 MB TIF)

Figure S6 Longitudinal distribution of earthworm gut bacteria with respect to time after feeding. Each panel represents the analysis of a single earthworm. The distribution of bacteria was determined by real-time PCR, quantifying the amount of the amount of the bacterial groups relative to total bacterial DNA (dissection as described in Fig. 1). Line colors represent the different bacterial groups as indicated in the figure.
(8.41 MB TIF)

Figure S7 Computer simulation of bacterial growth. The computer simulation was started with four different bacterial object types. The numbers of each object type is illustrated with the yellow, pink, and dark- and light-blue graphs. The first 200 generations simulate a starved situation, while the subsequent 200 generations simulate the situation after feeding. Details for the parameters used are given in Supplementary Materials and Methods.
(4.37 MB TIF)
