# Supplementary material for: A Feeding Induced Switch from a Variable to a Homogenous State of the Earthworm Gut Microbiota within a Host Population
Source: PLoS One. 2009 Oct 20;4(10):e7528. doi: 10.1371/journal.pone.0007528 (PMC2759579; doi:10.1371/journal.pone.0007528)
Supplement: Text S1 — (0.04 MB DOC) [file pone.0007528.s001.doc]

**TEXT S1**

**The microbiota associated with earthworms**

The first subset (n=333) represents a comparison between the microbiota in bedding/feed and the midgut of earthworms, while the other subset (n=667) represents the longitudinal distribution of bacteria in a single earthworm.

The first subset consisted of bacteria from bedding/feed (n=216) and dissected midgut samples (n=117; segment 3 in Fig. 1). Our major finding was that a bacterial group within *Proteobacteria* (quadrant 46, 31 in Fig. 2) was significantly overrepresented (p=0.005) in the midgut compared to the bedding/feed category. Blast searches of the GenBank non-redundant (nr) nucleotide sequence collection showed that this group resembled (99 % identity in the 16S rRNA gene) the denitrifying bacterium *Paracoccus denitrificans* [1]. We also identified a bacterial group significantly overrepresented (p=0.002) in the feed/bedding samples (quadrant 53, 30 in Fig. 2). We were not, however, able to identify close relatives of this group in the GenBank database by Blast search.

The second subset represents an analysis of the bacteria through a single whole earthworm collected prior to the experiment. This analysis was done to obtain an initial overview of the microbiota associated with earthworms before the main experiment. The dataset consisted of 8 categories representing the segments described in Figure 1. There was an overall dominance of bacteria (n = 601) with 98 – 100 % 16S rRNA gene identity to the genus *Acidiovorax* (determined by nr Blast search). These bacteria are probably symbionts in the nephridia of earthworms [2]. In total, there were 15 quadrants in Figure 2 containing ≥ 10 bacteria from the dataset. One of these quadrants (48, 1 in Fig. 2) showed a significant (p=0.05) overrepresentation in the fore- to midgut region (Suppl. Fig. 3). A GenBank Blast search with sequences from these bacteria showed approximately 98 % 16S rRNA gene identity to the actinobacterium *Leifsonia.* This genus has previously been associated with both plant pathogens [3] and growth on human implants [4].

**Computer simulations**

We used computer simulation to determine if the large variance in the microbiota in starved earthworms can be explained by the microbiota being in the non-equilibrium stochastic domain as defined by De Angelis and Waterhouse [5]. Since the niches in the earthworm gut are still undefined, we based our simulations on a hypothetical niche and four bacterial types. We are aware that this assumption certainly is not correct, but we used it to illustrate a principle.

We modeled the growth of individual bacteria as objects using the programming language C# in the .net programming environment (Microsoft Visual Studio.NET 2005, Microsoft Corp., Redmond, USA). We used a stochastic model for bacterial growth. Our model is based on calculating an index for whether a bacterium should divide or die. This index was based on the ratio between total number of bacterial objects and the number of objects of a given bacterial type. We included four bacterial types, allowing density-dependent internal competition in our model. Each bacterium was traversed once per generation. The decision of division or death was based on the following formula each time an object was traversed:

$$divsion\_death\_index=random\times\left(\frac{\sum bacteria}{\sum bacteria\_of\_same\_type+10^{5}}\right)$$

The *divison_death_index* is used to decide if a bacterium should divide or die, *random* is a random number between zero and one, *bacteria* represents each bacterial object, while *bacteria_of_same_type* represents an object of the same type as the given object.

We simulated 400 generations of bacterial growth. The first 200 generations simulated starvation with a *divison_death_index* < 0.001 for cell division, while the subsequent 200 generations simulated feeding with a *divison_death_index* < 0.1 for cell division. The consequence of a low *divison_death_index* is that the decision of cell division becomes independent of bacterial type, while for higher indexes the cell types become more predominant. In biological terms, an increase in the index means a shift from top-down towards bottom-up selection. Our model is based on the principle of parsimony, addressing the sole effect of population density at a “starved” level, and at a “fed” level. The intrinsic properties of all 4 bacterial object types are equal, and we simulate the competition within a single niche. A schematic outline of the simulation is shown in Supplementary Figure 1.

The starved situation in our simulation led to a community with an unstable coexistence of the four bacterial object types used in the simulation (Suppl. Fig. 7). After the simulated feeding, there was a rapid increase, between two and three log_10_, in the number of bacterial objects. In this situation, competitive exclusion led to a rapid shift to stable monocultures of bacterial objects within the simulated niche (Suppl. Fig. 7). The same pattern was also observed in two additional simulations (results not shown).

**SUPPLEMENTARY REFERENCES**

1. Baker SC, Ferguson SJ, Ludwig B, Page MD, Richter OM, et al. (1998) Molecular genetics of the genus Paracoccus: metabolically versatile bacteria with bioenergetic flexibility. Microbiol Mol Biol Rev 62: 1046-1078.

2. Schramm A, Davidson SK, Dodsworth JA, Drake HL, Stahl DA, et al. (2003) Acidovorax-like symbionts in the nephridia of earthworms. Environ Microbiol 5: 804-809.

3. Monteiro-Vitorello CB, Camargo LE, Van Sluys MA, Kitajima JP, Truffi D, et al. (2004) The genome sequence of the gram-positive sugarcane pathogen Leifsonia xyli subsp. xyli. Mol Plant Microbe Interact 17: 827-836.

4. Dempsey KE, Riggio MP, Lennon A, Hannah VE, Ramage G, et al. (2007) Identification of bacteria on the surface of clinically infected and non-infected prosthetic hip joints removed during revision arthroplasties by 16S rRNA gene sequencing and by microbiological culture. Arthritis Res Ther 9: R46.

5. DeAngelis DL, Waterhouse JC (1987) Equilibrium and nonequilibrium concepts in ecological models. Ecological Monographs 57: 1-21.
