# Supplementary material for: A Feeding Induced Switch from a Variable to a Homogenous State of the Earthworm Gut Microbiota within a Host Population
Source: PLoS One. 2009 Oct 20;4(10):e7528. doi: 10.1371/journal.pone.0007528 (PMC2759579; doi:10.1371/journal.pone.0007528)
Supplement: Table S1 — RDPII hierarchical classification of 16S rRNA (0.23 MB DOC) [file pone.0007528.s002.doc]

**Table S1. RDPII hierarchical classification of 16S rRNA^1^**

| domain Bacteria (998) |  |  |  |  |  |  |  |
| --- | --- | --- | --- | --- | --- | --- | --- |
|  | phylum Planctomycetes (1) |  |  |  |  |  |  |
|  |  | class Planctomycetacia (1) |  |  |  |  |  |
|  |  |  | order Planctomycetales (1) |  |  |  |  |
|  |  |  |  | family Planctomycetaceae (1) |  |  |  |
|  |  |  |  |  | genus Isosphaera (1) |  |  |
|  | phylum Verrucomicrobia (2) |  |  |  |  |  |  |
|  |  | class Verrucomicrobiae (2) |  |  |  |  |  |
|  |  |  | order Verrucomicrobiales (2) |  |  |  |  |
|  |  |  |  | unclassified_Verrucomicrobiales (2) |  |  |  |
|  | phylum Firmicutes (5) |  |  |  |  |  |  |
|  |  | class Bacilli (1) |  |  |  |  |  |
|  |  |  | order Bacillales (1) |  |  |  |  |
|  |  |  |  | unclassified_Bacillales (1) |  |  |  |
|  |  | class Clostridia (4) |  |  |  |  |  |
|  |  |  | order Clostridiales (3) |  |  |  |  |
|  |  |  |  | family Clostridiaceae (2) |  |  |  |
|  |  |  |  |  | genus Acetivibrio (1) |  |  |
|  |  |  |  |  | genus Clostridium (1) |  |  |
|  |  |  |  | unclassified_Clostridiales (1) |  |  |  |
|  |  |  | unclassified_Clostridia (1) |  |  |  |  |
|  | phylum Genera_incertae_sedis_TM7 (3) |  |  |  |  |  |  |
|  |  | genus TM7 (3) |  |  |  |  |  |
|  | phylum Bacteroidetes (97) |  |  |  |  |  |  |
|  |  | class Flavobacteria (28) |  |  |  |  |  |
|  |  |  | order Flavobacteriales (28) |  |  |  |  |
|  |  |  |  | family Cryomorphaceae (1) |  |  |  |
|  |  |  |  |  | genus Brumimicrobium (1) |  |  |
|  |  |  |  | family Flavobacteriaceae (27) |  |  |  |
|  |  |  |  |  | genus Flavobacterium (24) |  |  |
|  |  |  |  |  | unclassified_Flavobacteriaceae (3) |  |  |
|  |  | class Sphingobacteria (60) |  |  |  |  |  |
|  |  |  | order Sphingobacteriales (60) |  |  |  |  |
|  |  |  |  | family Sphingobacteriaceae (7) |  |  |  |
|  |  |  |  |  | genus Pedobacter (7) |  |  |
|  |  |  |  | family Flexibacteraceae (37) |  |  |  |
|  |  |  |  |  | genus Sporocytophaga (1) |  |  |
|  |  |  |  |  | genus Dyadobacter (1) |  |  |
|  |  |  |  |  | unclassified_Flexibacteraceae (35) |  |  |
|  |  |  |  | family Crenotrichaceae (2) |  |  |  |
|  |  |  |  |  | genus Chitinophaga (2) |  |  |
|  |  |  |  | unclassified_Sphingobacteriales (14) |  |  |  |
|  |  | unclassified_Bacteroidetes (9) |  |  |  |  |  |
|  | phylum Actinobacteria (134) |  |  |  |  |  |  |
|  |  | class Actinobacteria (134) |  |  |  |  |  |
|  |  |  | subclass Rubrobacteridae (2) |  |  |  |  |
|  |  |  |  | order Rubrobacterales (2) |  |  |  |
|  |  |  |  |  | suborder Rubrobacterineae (2) |  |  |
|  |  |  |  |  |  | family Rubrobacteraceae (2) |  |
|  |  |  |  |  |  |  | genus Conexibacter (1) |
|  |  |  |  |  |  |  | unclassified_Rubrobacteraceae (1) |
|  |  |  | subclass Actinobacteridae (132) |  |  |  |  |
|  |  |  |  | order Actinomycetales (132) |  |  |  |
|  |  |  |  |  | suborder Pseudonocardineae (2) |  |  |
|  |  |  |  |  |  | family Pseudonocardiaceae (2) |  |
|  |  |  |  |  |  |  | genus Pseudonocardia (1) |
|  |  |  |  |  |  |  | genus Saccharopolyspora (1) |
|  |  |  |  |  | suborder Corynebacterineae (2) |  |  |
|  |  |  |  |  |  | family Corynebacteriaceae (2) |  |
|  |  |  |  |  |  |  | genus Corynebacterium (2) |
|  |  |  |  |  | suborder Propionibacterineae (32) |  |  |
|  |  |  |  |  |  | family Propionibacteriaceae (6) |  |
|  |  |  |  |  |  |  | genus Propionibacterium (5) |
|  |  |  |  |  |  |  | genus Microlunatus (1) |
|  |  |  |  |  |  | family Nocardioidaceae (25) |  |
|  |  |  |  |  |  |  | genus Aeromicrobium (1) |
|  |  |  |  |  |  |  | genus Kribbella (10) |
|  |  |  |  |  |  |  | genus Nocardioides (11) |
|  |  |  |  |  |  |  | unclassified_Nocardioidaceae (3) |
|  |  |  |  |  |  | unclassified_Propionibacterineae (1) |  |
|  |  |  |  |  | suborder Streptosporangutneae (1) |  |  |
|  |  |  |  |  |  | family Streptosporangutaceae (1) |  |
|  |  |  |  |  |  |  | unclassified_Streptosporangutaceae (1) |
|  |  |  |  |  | suborder Streptomycineae (24) |  |  |
|  |  |  |  |  |  | family Streptomycetaceae (24) |  |
|  |  |  |  |  |  |  | genus Streptomyces (19) |
|  |  |  |  |  |  |  | unclassified_Streptomycetaceae (5) |
|  |  |  |  |  | suborder Micrococcineae (51) |  |  |
|  |  |  |  |  |  | family Promicromonosporaceae (1) |  |
|  |  |  |  |  |  |  | genus Promicromonospora (1) |
|  |  |  |  |  |  | family Microbacteriaceae (43) |  |
|  |  |  |  |  |  |  | genus Agromyces (1) |
|  |  |  |  |  |  |  | genus Microbacterium (4) |
|  |  |  |  |  |  |  | genus Leifsonia (3) |
|  |  |  |  |  |  |  | unclassified_Microbacteriaceae (35) |
|  |  |  |  |  |  | family Intrasporangutaceae (2) |  |
|  |  |  |  |  |  |  | unclassified_Intrasporangutaceae (2) |
|  |  |  |  |  |  | family Micrococcaceae (2) |  |
|  |  |  |  |  |  |  | genus Arthrobacter (1) |
|  |  |  |  |  |  |  | unclassified_Micrococcaceae (1) |
|  |  |  |  |  |  | family Sanguibacteraceae (1) |  |
|  |  |  |  |  |  |  | genus Sanguibacter (1) |
|  |  |  |  |  |  | unclassified_Micrococcineae (2) |  |
|  |  |  |  |  | unclassified_Actinomycetales (20) |  |  |
|  | phylum Proteobacteria (705) |  |  |  |  |  |  |
|  |  | class Alphaproteobacteria (56) |  |  |  |  |  |
|  |  |  | order Rhodobacterales (26) |  |  |  |  |
|  |  |  |  | family Rhodobacteraceae (26) |  |  |  |
|  |  |  |  |  | genus Paracoccus (21) |  |  |
|  |  |  |  |  | unclassified_Rhodobacteraceae (5) |  |  |
|  |  |  | order Rickettsiales (1) |  |  |  |  |
|  |  |  |  | unclassified_Rickettsiales (1) |  |  |  |
|  |  |  | order Sphingomonadales (4) |  |  |  |  |
|  |  |  |  | family Sphingomonadaceae (4) |  |  |  |
|  |  |  |  |  | genus Novosphingobium (3) |  |  |
|  |  |  |  |  | genus Sphingomonas (1) |  |  |
|  |  |  | order Caulobacterales (8) |  |  |  |  |
|  |  |  |  | family Caulobacteraceae (8) |  |  |  |
|  |  |  |  |  | genus Phenylobacterium (1) |  |  |
|  |  |  |  |  | genus Brevundimonas (1) |  |  |
|  |  |  |  |  | genus Caulobacter (5) |  |  |
|  |  |  |  |  | unclassified_Caulobacteraceae (1) |  |  |
|  |  |  | order Rhizobiales (13) |  |  |  |  |
|  |  |  |  | family Rhizobiaceae (2) |  |  |  |
|  |  |  |  |  | genus Rhizobium (1) |  |  |
|  |  |  |  |  | unclassified_Rhizobiaceae (1) |  |  |
|  |  |  |  | family Brucellaceae (1) |  |  |  |
|  |  |  |  |  | unclassified_Brucellaceae (1) |  |  |
|  |  |  |  | family Hyphomicrobiaceae (1) |  |  |  |
|  |  |  |  |  | unclassified_Hyphomicrobiaceae (1) |  |  |
|  |  |  |  | family Methylobacteriaceae (4) |  |  |  |
|  |  |  |  |  | genus Methylobacterium (4) |  |  |
|  |  |  |  | unclassified_Rhizobiales (5) |  |  |  |
|  |  |  | unclassified_Alphaproteobacteria (4) |  |  |  |  |
|  |  | class Betaproteobacteria (603) |  |  |  |  |  |
|  |  |  | order Hydrogenophilales (2) |  |  |  |  |
|  |  |  |  | family Hydrogenophilaceae (2) |  |  |  |
|  |  |  |  |  | genus Hydrogenophilus (1) |  |  |
|  |  |  |  |  | genus Petrobacter (1) |  |  |
|  |  |  | order Burkholderiales (601) |  |  |  |  |
|  |  |  |  | family Alcaligenaceae (1) |  |  |  |
|  |  |  |  |  | genus Achromobacter (1) |  |  |
|  |  |  |  | family Comamonadaceae (269) |  |  |  |
|  |  |  |  |  | unclassified_Comamonadaceae (269) |  |  |
|  |  |  |  | family Incertae sedis 5 (1) |  |  |  |
|  |  |  |  |  | unclassified_Incertae sedis 5 (1) |  |  |
|  |  |  |  | unclassified_Burkholderiales (330) |  |  |  |
|  |  | class Gammaproteobacteria (42) |  |  |  |  |  |
|  |  |  | order Aeromonadales (6) |  |  |  |  |
|  |  |  |  | family Aeromonadaceae (6) |  |  |  |
|  |  |  |  |  | genus Aeromonas (6) |  |  |
|  |  |  | order Legutonellales (1) |  |  |  |  |
|  |  |  |  | family Legutonellaceae (1) |  |  |  |
|  |  |  |  |  | genus Legutonella (1) |  |  |
|  |  |  | order Enterobacteriales (5) |  |  |  |  |
|  |  |  |  | family Enterobacteriaceae (5) |  |  |  |
|  |  |  |  |  | genus Raoultella (1) |  |  |
|  |  |  |  |  | unclassified_Enterobacteriaceae (4) |  |  |
|  |  |  | order Xanthomonadales (4) |  |  |  |  |
|  |  |  |  | family Xanthomonadaceae (4) |  |  |  |
|  |  |  |  |  | genus Lysobacter (1) |  |  |
|  |  |  |  |  | unclassified_Xanthomonadaceae (3) |  |  |
|  |  |  | order Pseudomonadales (19) |  |  |  |  |
|  |  |  |  | family Incertae sedis 6 (2) |  |  |  |
|  |  |  |  |  | genus Enhydrobacter (2) |  |  |
|  |  |  |  | family Moraxellaceae (1) |  |  |  |
|  |  |  |  |  | genus Acinetobacter (1) |  |  |
|  |  |  |  | family Pseudomonadaceae (16) |  |  |  |
|  |  |  |  |  | genus Pseudomonas (11) |  |  |
|  |  |  |  |  | genus Cellvibrio (3) |  |  |
|  |  |  |  |  | unclassified_Pseudomonadaceae (2) |  |  |
|  |  |  | unclassified_Gammaproteobacteria (7) |  |  |  |  |
|  |  | unclassified_Proteobacteria (4) |  |  |  |  |  |
|  | unclassified_Bacteria (51) |  |  |  |  |  |  |
| unclassified_Root (2) |  |  |  |  |  |  |  |

^1^ The numbers in the parenthesis indicate the number of clones.
