# Supplementary figures and images for: A Feeding Induced Switch from a Variable to a Homogenous State of the Earthworm Gut Microbiota within a Host Population
Source: PLoS One. 2009 Oct 20;4(10):e7528. doi: 10.1371/journal.pone.0007528 (PMC2759579; doi:10.1371/journal.pone.0007528)

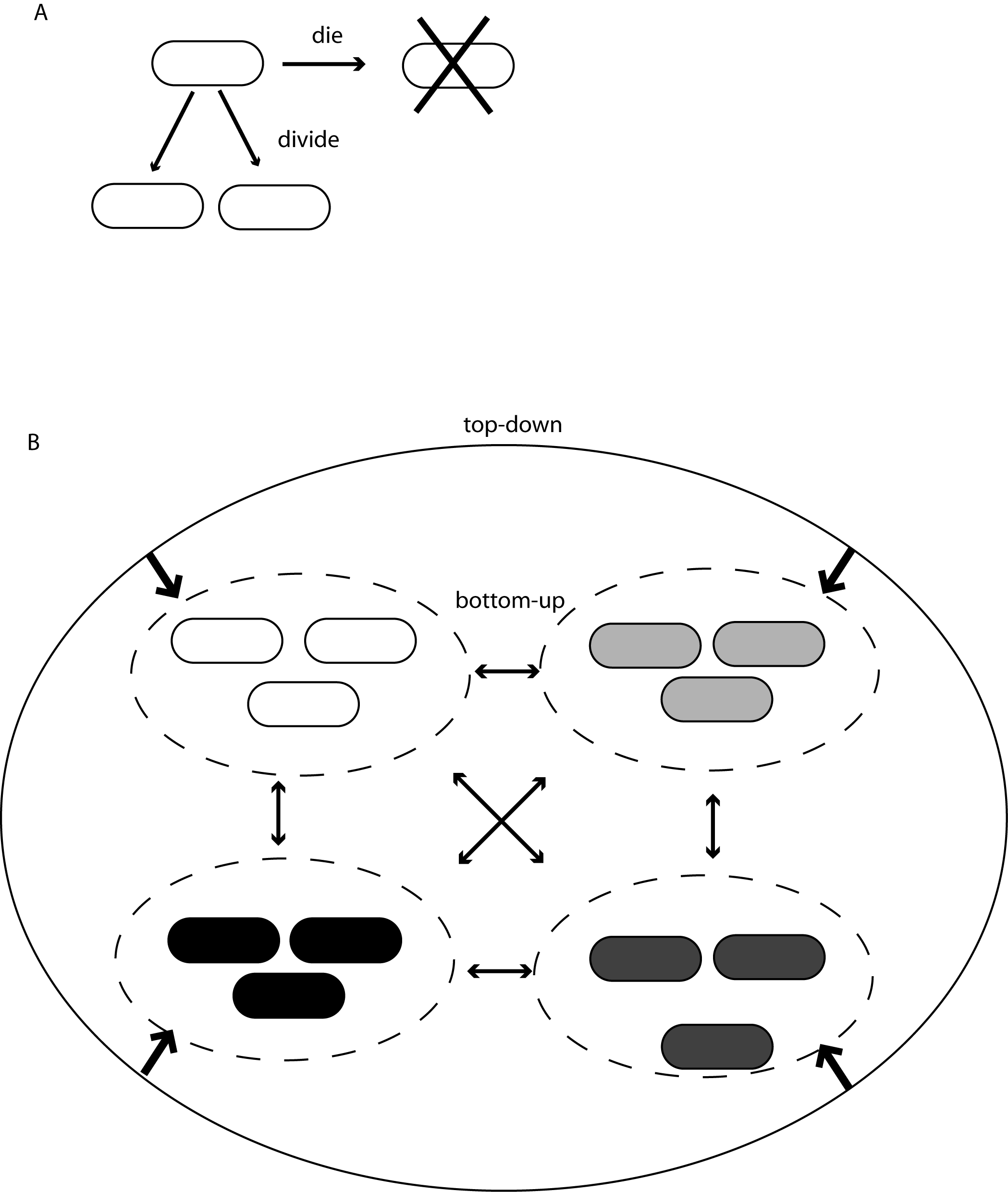

Supplement: Figure S1 — Model for computer simulation of bacterial growth. (A) For each generation, the bacterial objects can either divide or die. (B) The decision of division or death is based on cell density-dependent internal competition (bottom-up) of four bacterial groups occupying the same niche. The total cell density within the niche is limited by external factors (top-down). (5.22 MB TIF) [file pone.0007528.s003.tif]

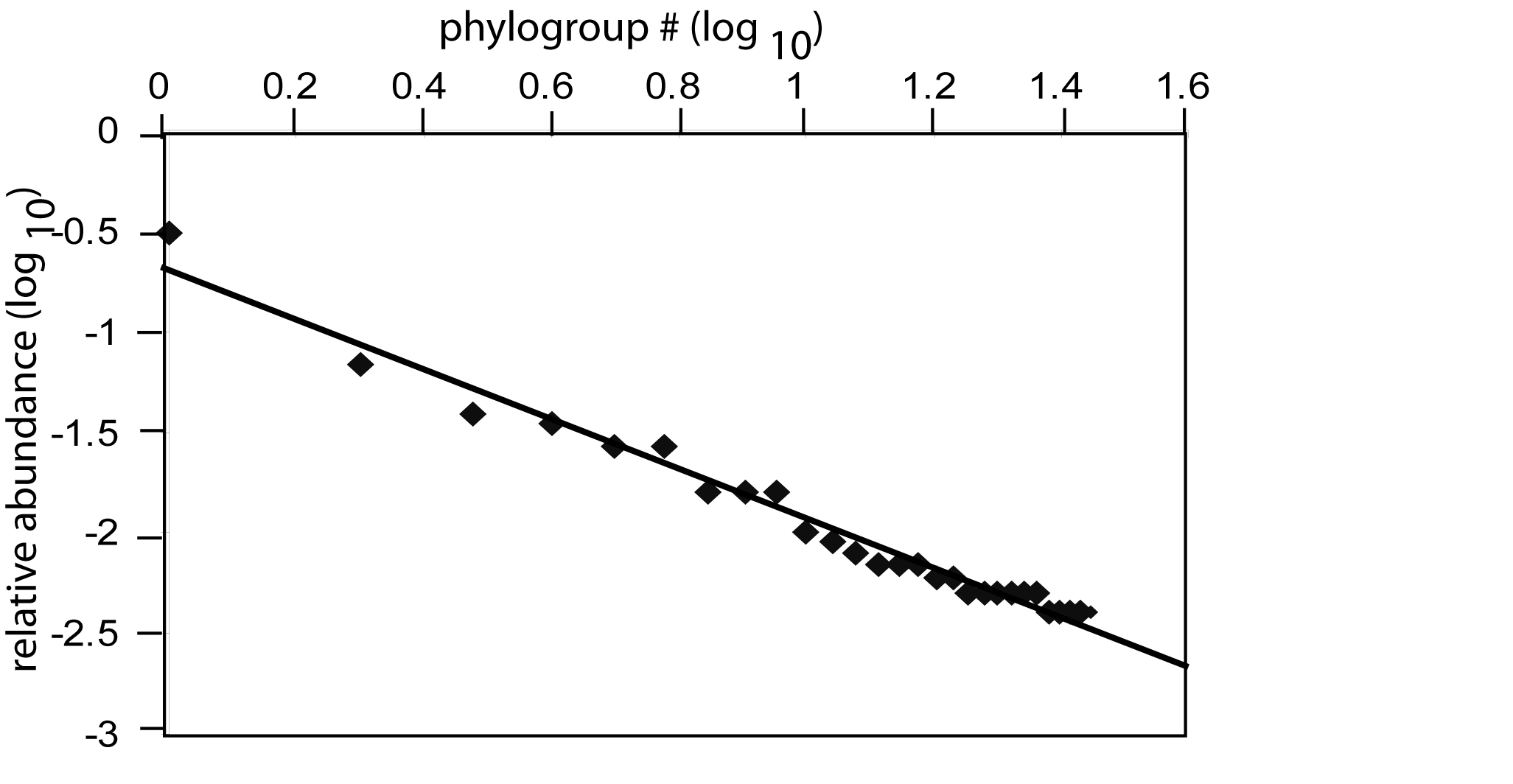

Supplement: Figure S2 — Density distribution curve for the earthworm-associated microbiota. The log10 of the relative abundance of the phylogroups (squares in Fig. 2) is plotted as a scatter plot with respect to the log10 of the range of the phylogroups. Only phylogroups with a abundance of n = 4 or higher are included due to the reliability of the density determinations. The formula for the regression line is as follows: Abundance (log10) = −1.2×Range (log10)−0.7, R2 = 0.98. (2.20 MB TIF) [file pone.0007528.s004.tif]

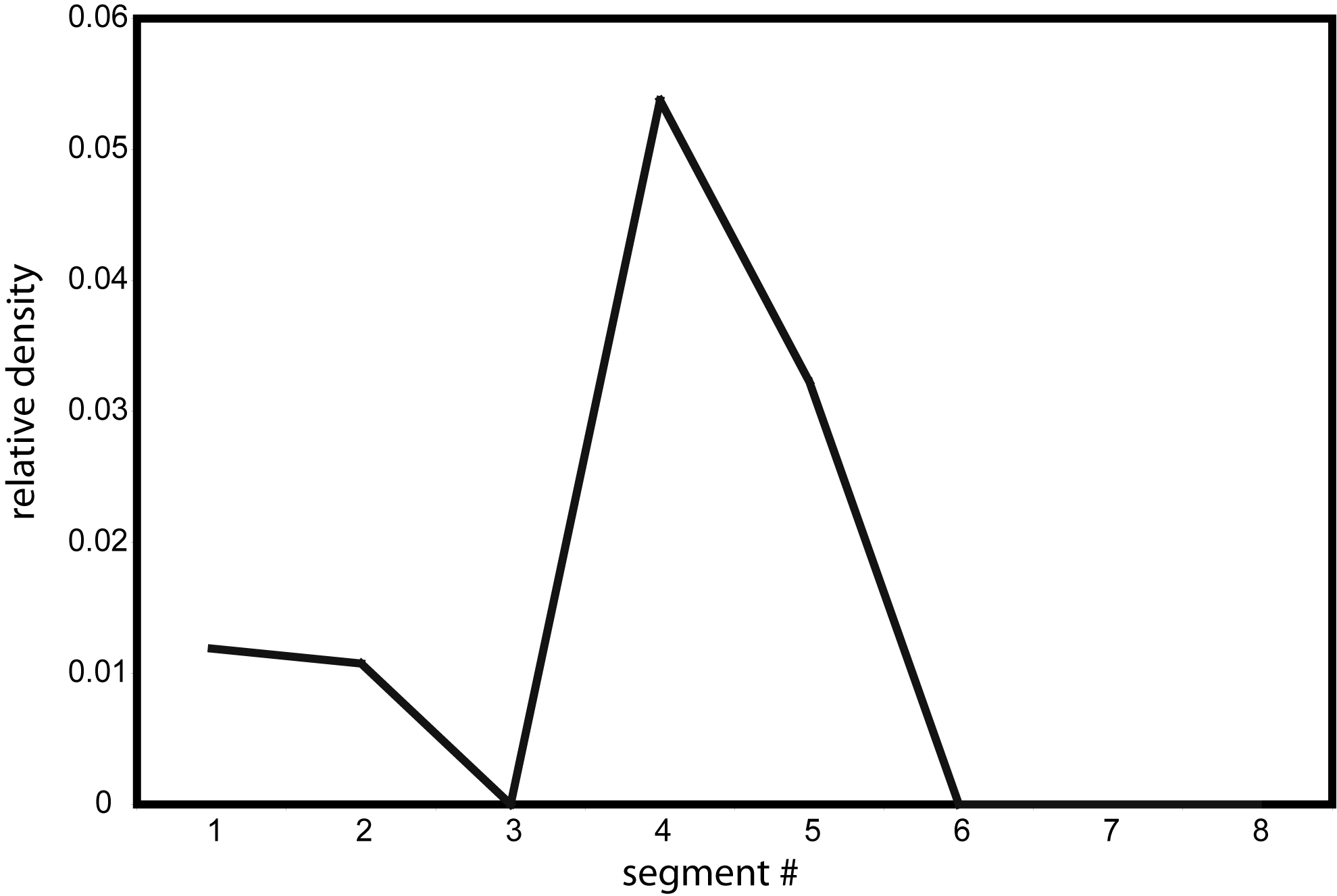

Supplement: Figure S3 — Longitudinal distribution of a bacterial group significantly overrepresented in the earthworm midgut region. The group is defined by the coordinates 48, 1 in Figure 2, and was overrepresented at the p = 0.05 level. The relative distributions in the eight segments analyzed (see Fig. 1 for reference) are shown. (2.64 MB TIF) [file pone.0007528.s005.tif]

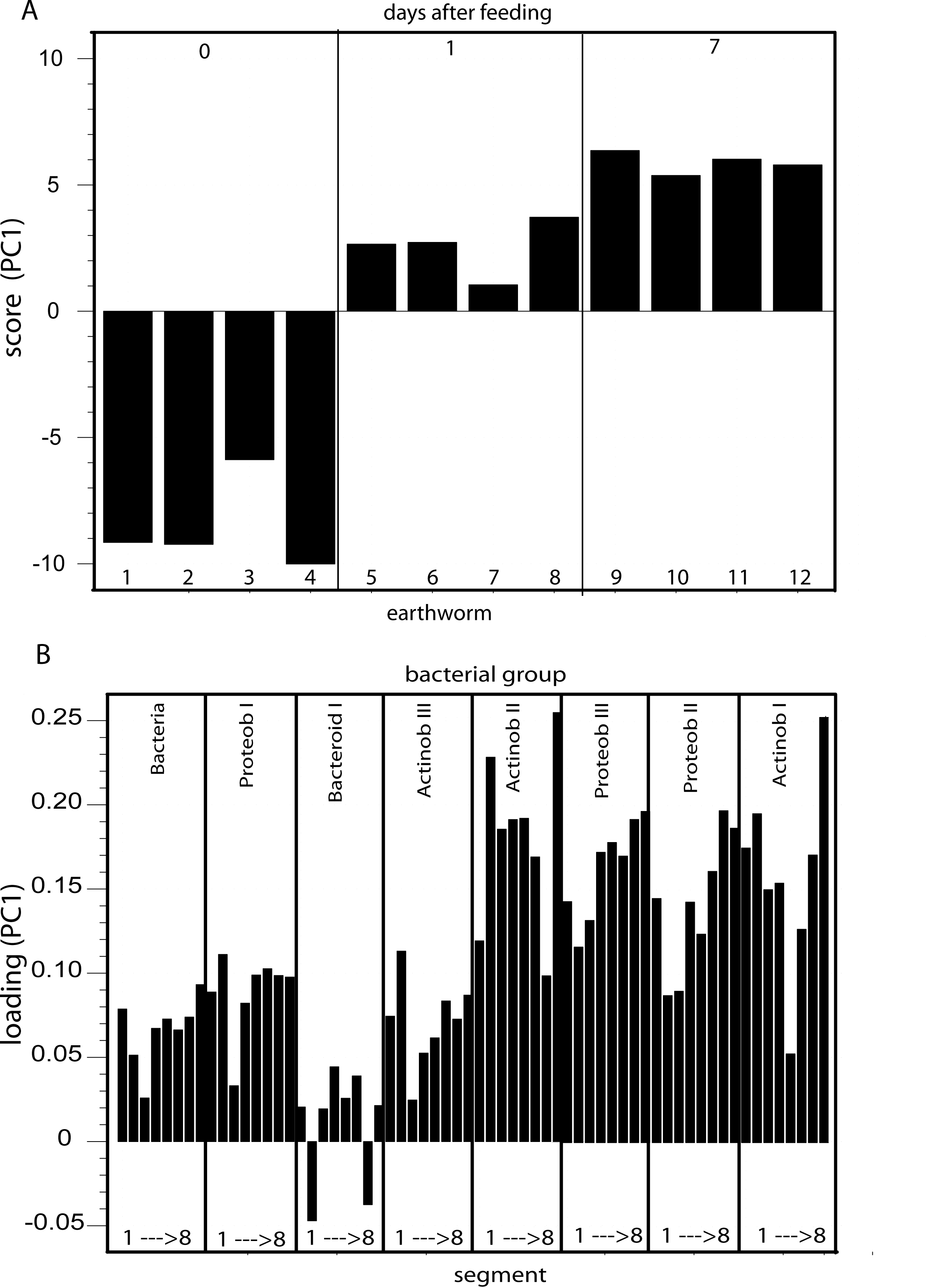

Supplement: Figure S4 — Regression between days after feeding and longitudinal distribution of bacteria in earthworms. The regression is based on the real-time quantitative PCR data. Results for the first PC are shown, explaining 70% of the variance for the bacterial groups and 58% of the variance for days after feeding data. (A) A score plot showing the relatedness in the microbiota with respect to days after feeding. (B) A loading plot showing which bacterial groups and segments that are important for explaining the overall pattern shown in panel A. The numbers 1 → 8 refers to the segments analyzed. (7.93 MB TIF) [file pone.0007528.s006.tif]

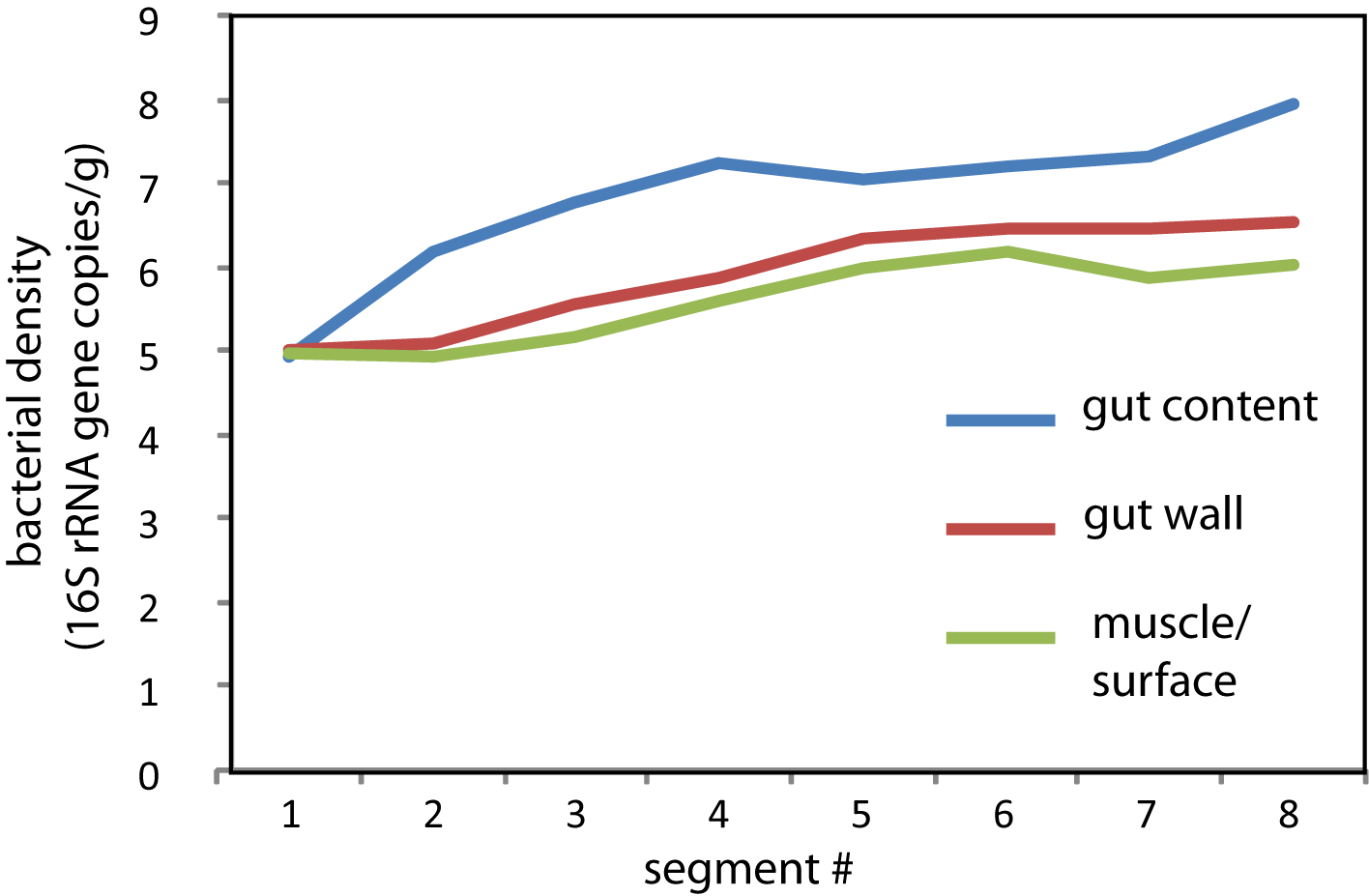

Supplement: Figure S5 — Spatial distribution of earthworm bacteria. The spatial distribution of bacteria was determined one day after feeding for a single earthworm. Each of the eight segments for the whole earthworm analyses were dissected into three samples: surface/muscle, gut wall and gut content. The quantification of bacteria is expressed relative to the weight of the material analyzed using the Bacteria primer pair. (3.96 MB TIF) [file pone.0007528.s007.tif]

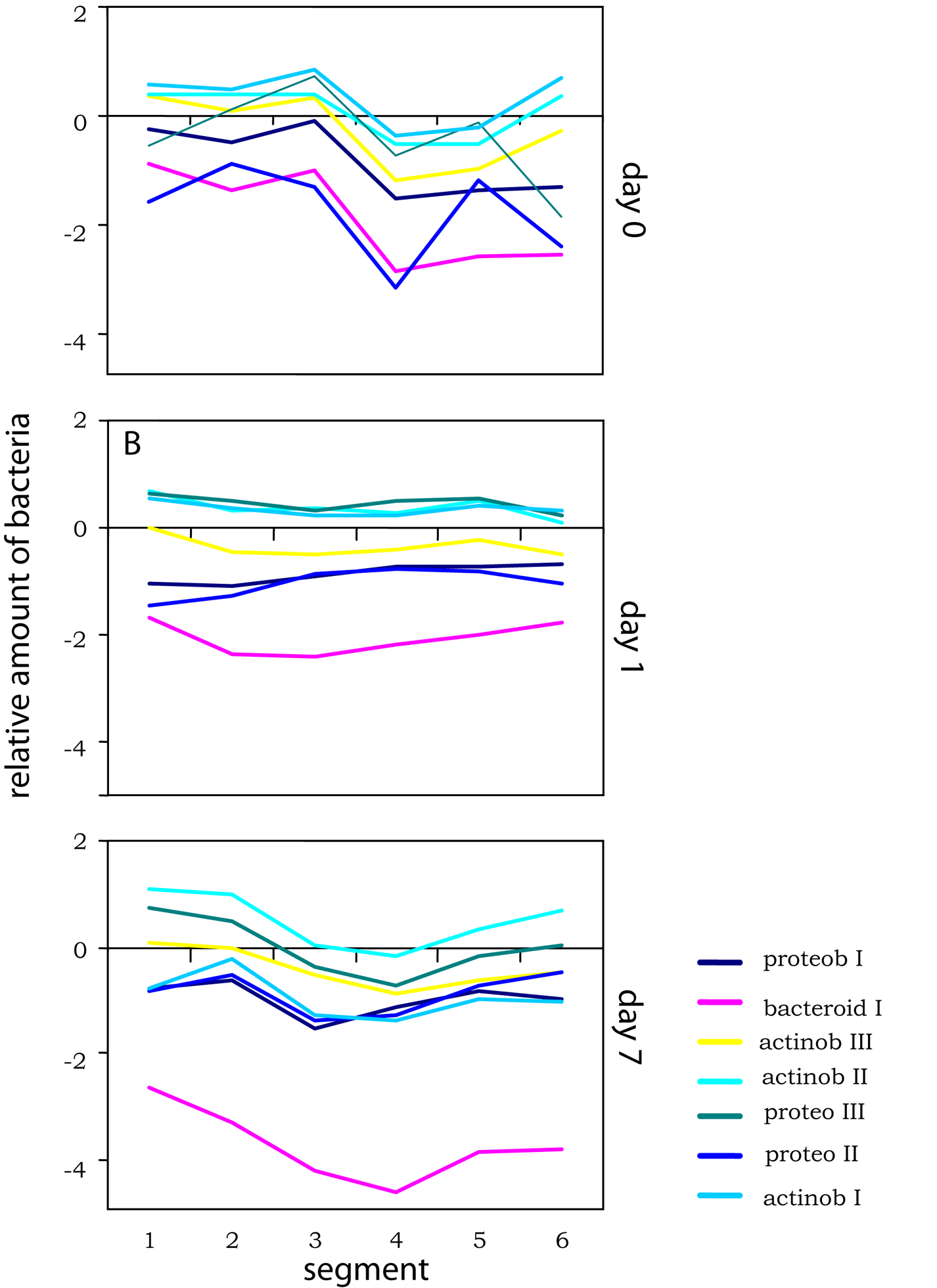

Supplement: Figure S6 — Longitudinal distribution of earthworm gut bacteria with respect to time after feeding. Each panel represents the analysis of a single earthworm. The distribution of bacteria was determined by real-time PCR, quantifying the amount of the amount of the bacterial groups relative to total bacterial DNA (dissection as described in Fig. 1). Line colors represent the different bacterial groups as indicated in the figure. (8.41 MB TIF) [file pone.0007528.s008.tif]

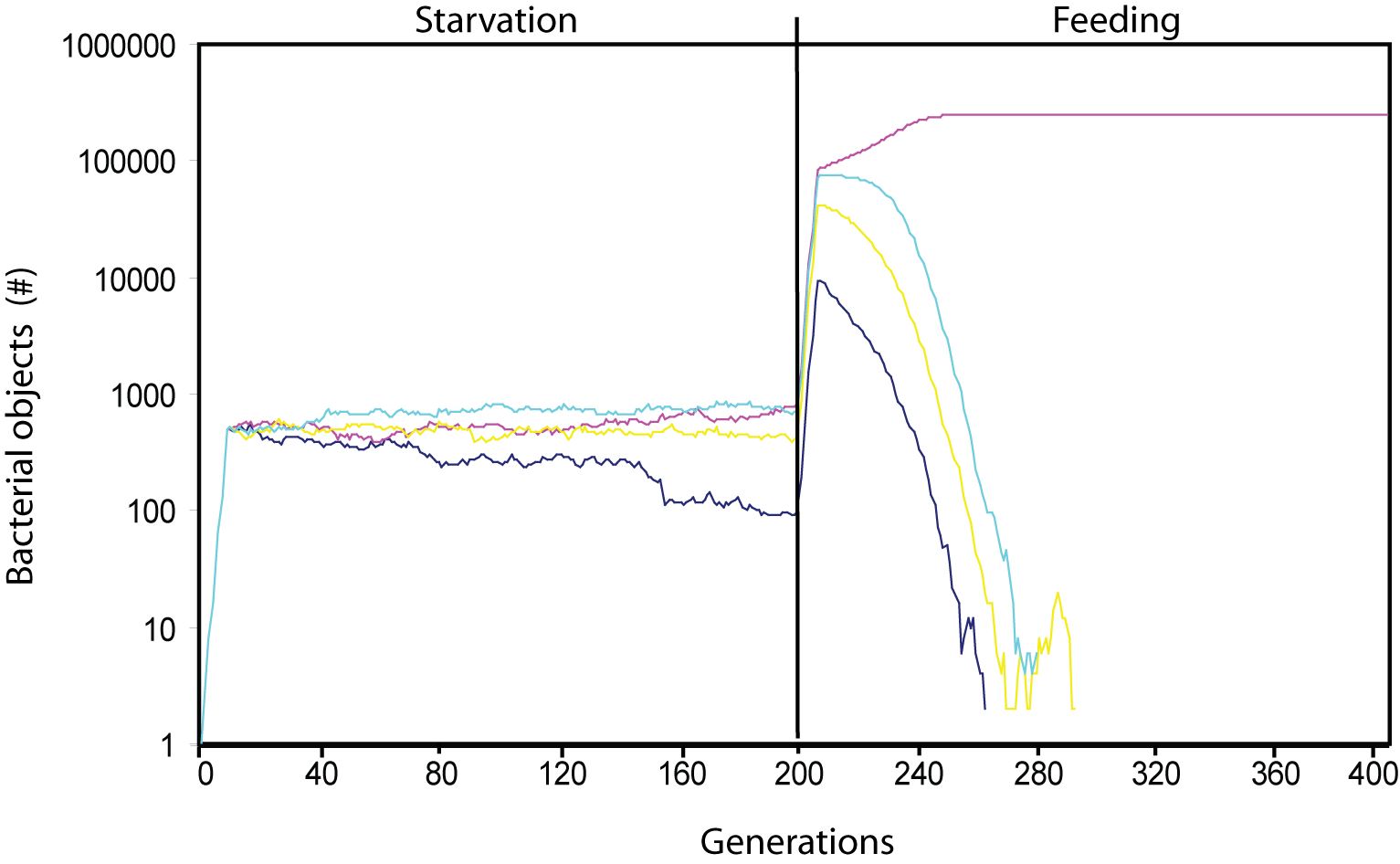

Supplement: Figure S7 — Computer simulation of bacterial growth. The computer simulation was started with four different bacterial object types. The numbers of each object type is illustrated with the yellow, pink, and dark- and light-blue graphs. The first 200 generations simulate a starved situation, while the subsequent 200 generations simulate the situation after feeding. Details for the parameters used are given in Supplementary Materials and Methods. (4.37 MB TIF) [file pone.0007528.s009.tif]
